# Root‐Inspired Microneedle Patch With Interlocked Rigid‐Soft Architecture for High Tissue‐Adaptiveness

**DOI:** 10.1002/advs.77211

**Published:** 2026-08-15

**Authors:** Jongchan Lee, Yeongjun Song, Yonghyun Cho, Hyo In Kim, Dong‐Hyun Youn, Jinbong Park, Seungbeom Choi, Dohyun Kwon, Brian Jun Lee, Sangyul Baik

**Affiliations:** ^1^ Department of Mechanical Engineering Sungkyunkwan University Suwon South Korea; ^2^ Division of Pharmacology Pusan National University Yangsan South Korea; ^3^ Department of Pharmacology Kyunghee University Seoul South Korea; ^4^ Department of Oral and Maxillofacial Surgery Samsung Medical Center Seoul South Korea

**Keywords:** additive manufacturing, biomimetics, drug delivery, microneedle, tissue adhesion

## Abstract

Microneedle technology offers a minimally invasive, pain‐free platform for drug delivery with high patient compliance and dosing precision. However, these systems struggle to adhere to curved, moist biological surfaces with dynamic movements, which require flexible substrates for conformity and rigid needles for penetration, resulting in a fundamental mechanical mismatch. Here, we present a root‐inspired microneedle array patch (R‐MAP) that integrates rigid microneedles and flexible substrates via lattice‐based mechanical interlocking. Peel‐off tests revealed a three‐fold increase in interfacial bonding force compared to non‐interlocked controls. Ex vivo evaluations confirmed stable penetration and reliable adhesion under dynamic conditions. To further enhance wet‐surface retention, we introduced a hydrogel adhesion layer, enabling in vivo attachment to porcine oral mucosa for over 5 h without detachment despite extreme rubbing conditions. This work provides a structurally robust, adaptable solution for challenging drug delivery environments, expanding the applicability of microneedles to curved, moist surfaces with dynamic movements including oral and dermal tissues.

## Graphical abstract

A root‐inspired microneedle array patch uses lattice‐based mechanical interlocking to unite rigid microneedles with a flexible substrate. The interlocked rigid–soft architecture improves interfacial bonding, supports stable adhesion on curved and wet tissues under dynamic motion, and provides a versatile platform for robust biointerfacing and future rigid device integration.

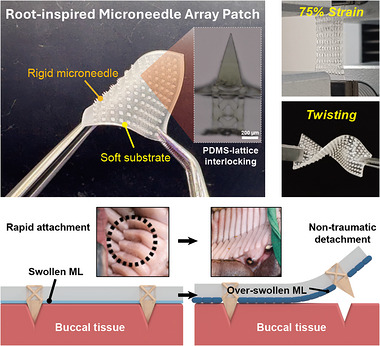

## 1 Introduction

Microneedle (MN) technology has emerged as a transformative platform for drug delivery applications, promising [1] avoidance of gastrointestinal degradation and hepatic first‐pass metabolism [2]. Furthermore, MNs enable controlled and precise dosing [3, 4], significantly improving therapeutic efficacy compared to conventional topical formulations such as gels or creams [5]. Despite these advantages, current MN‐based drug delivery systems face significant hurdles in achieving effective and sustained attachment to biological surfaces, particularly skin or mucosal tissues that are inherently complex, curved, and dynamically moving. This challenge primarily arises from a mechanical modulus mismatch [6, 7, 8, 9] between the rigid microneedles, necessary for tissue penetration, and flexible substrates required to conform to these irregular surfaces. For instance, rigid MN arrays fabricated through conventional injection molding or 3D printing typically exhibit limited adaptability to skin or mucosal curvatures, leading to compromised adhesion and inconsistent drug absorption [10]. Conversely, flexible MN arrays produced from porous materials or hydrogels exhibit superior conformity but lack sufficient mechanical strength for effective tissue penetration. Thus, overcoming this rigidity‐flexibility trade‐off is essential for advancing MN‐based drug delivery technologies.

Various approaches have been explored to simultaneously achieve robust MN penetration and patch flexibility. Multi‐material 3D printing techniques have been introduced to fabricate MN arrays, combining materials of different stiffnesses in a single step, aiming to balance the required rigidity and flexibility [11]. However, this approach is limited by narrow ranges of compatible materials and potential structural weaknesses at the interface between materials with highly distinct mechanical properties. Alternative studies utilized chemical adhesion methods, attaching rigid MN arrays made of epoxy siloxane or similar hard materials onto flexible polymer substrates such as polydimethylsiloxane (PDMS) [12]. However, chemically bonded microneedle devices frequently exhibit weak interfacial adhesion and structural failure due to substantial differences in surface free energy and mechanical modulus between rigid needles and soft substrates [13]. Flexible substrates such as PDMS are chemically inert with low surface energies, making it challenging to establish robust adhesive bonds with rigid materials. Furthermore, differences in elastic moduli result in incompatible deformation under mechanical loads, leading to intense stress concentrations at material interfaces, facilitating interfacial delamination or needle detachment. Conventional chemical adhesion methods like epoxy bonding, though initially strong, are brittle and incapable of accommodating differential strain, further exacerbating interfacial failures. Thus, there is an urgent need for innovative fabrication methods capable of mechanically and reliably coupling rigid microneedles to flexible substrates without relying solely on chemical adhesion.

**FIGURE 1:**
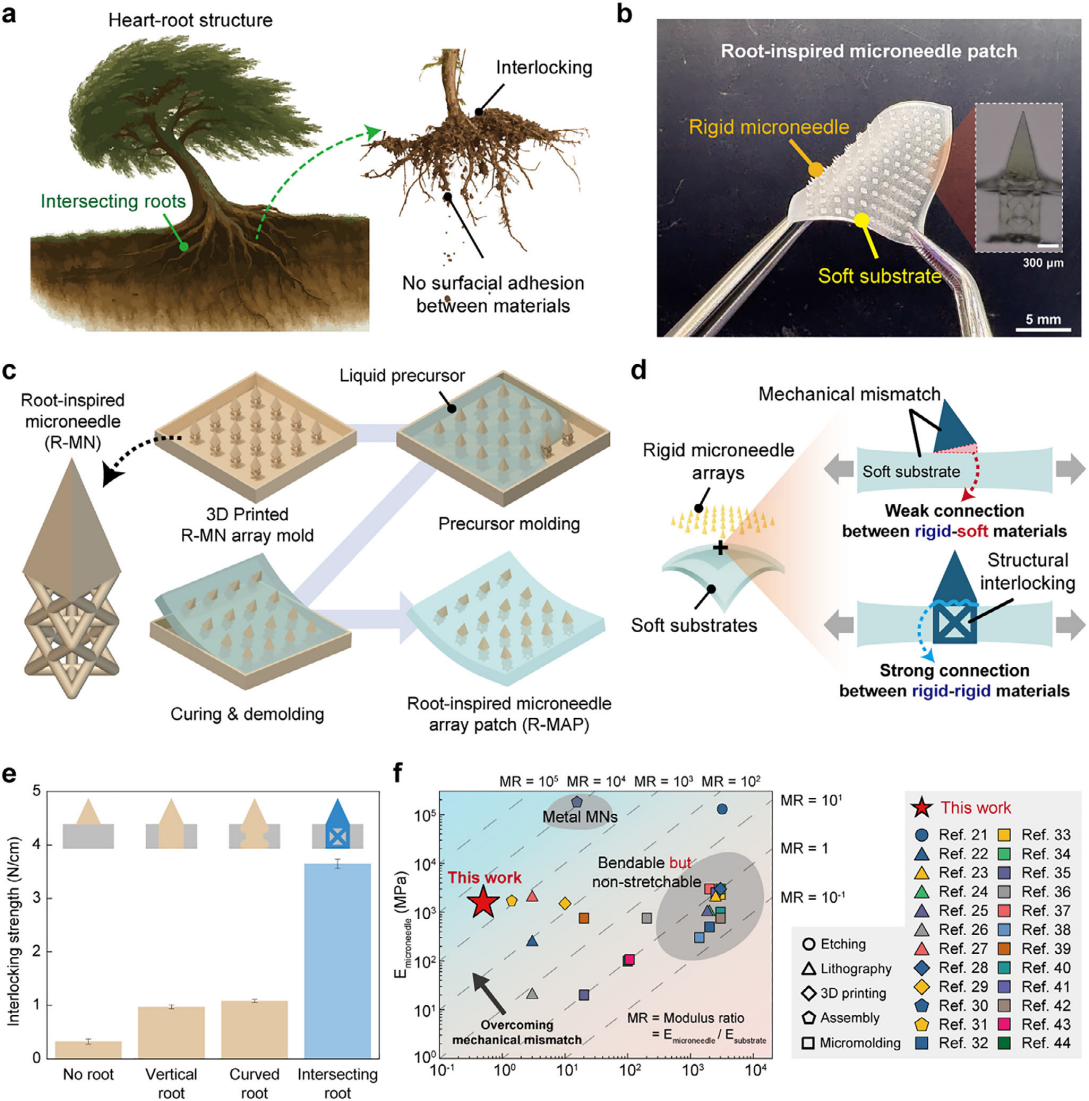
(a) Heart root‐soil interlocking between two materials with mechanical mismatch. (b) Photograph of R‐MAP. The inset shows a microscope image of the root‐inspired microneedle. (c) Schematic illustration of the design and manufacturing process of R‐MAP. (d) Limitation of flexible patches constructed through interfacial bonding between two mechanically mismatched materials, and the R‐MAP mechanism for overcoming mechanical mismatch through structural interlocking. (e) Interlocking force comparison of various nature‐inspired supporting structures (*n* = 3). (f) Comparison of flexible microneedle systems based on modulus mismatch ratio.

Nature presents a simple and innovative solution to this problem by offering evolved architectures that inherently overcome mechanical mismatch. Heart‐root systems, as observed in species such as oak trees, feature thick roots extending in vertical, horizontal, and oblique directions, forming a multi‐axial network that maximizes the contact area between roots and soil [14, 15]. This geometry not only enables exceptionally high pull‐out resistance but also ensures strong lateral anchorage by distributing mechanical loads across roots oriented in multiple directions [16]. When embedded in clay‐rich soils, these root systems engage in mechanical interlocking with the surrounding matrix, effectively transforming the root–soil interface into a unified anchoring structure [17]. As a result, trees maintain structural stability and withstand destabilizing forces such as soil saturation or wind stress on sloped terrains [18, 19] (Figure 1a).

Inspired by this natural root interlocking mechanism, here we introduce a root‐inspired microneedle array patch (R‐MAP) that mechanically couples dissimilar materials through lattice interlocking structures (Figure 1b). Utilizing advanced 3D printing techniques, rigid lattice‐based microneedle arrays and flexible PDMS substrates can be integratively assembled without relying on chemical adhesives, creating a robust interlocking interface capable of accommodating two totally different materials with drastically different elastic moduli within a single device. To validate the mechanical stability and applicability of our proposed mechanical interlocking approach, we conducted peel‐off tests to quantitatively assess the adhesion strength between rigid microneedle arrays and flexible substrates. Additionally, the flexibility of patches was systematically characterized by measuring bending stresses across varying lattice configurations. The mechanical robustness of the interlocked microneedle patches was further evaluated through penetration tests, confirming sufficient structural integrity for effective insertion into target tissues. Ex vivo experiments demonstrated consistent penetration performance and reliable adhesion capabilities. To further enhance the practical applicability under moist conditions, we incorporated an additional hydrogel adhesion layer, significantly improving wet surface adhesion stability. To demonstrate the applicability of this approach, we focused on the oral mucosa as a target site, since oral mucosa is an ideal administration route due to its high vascularization, substantial permeability, and direct systemic access, enabling rapid onset of therapeutic action [20]. We present a miniaturized device inspired by human oral movements to investigate stable mounting within the oral vestibule under harsh frictional conditions. In vivo studies in a miniature‐pig model verified that our R‐MAP could reliably maintain adhesion in an extremely harsh environment, highlighting its substantial potential as a transformative solution for oral transmucosal drug delivery systems. This novel approach simultaneously addresses the critical issues of robust mucosal penetration, adaptability to complex surfaces, and prolonged adhesion under curved, moist conditions with dynamic movements.

## 2 Results

### 2.1 Design and Manufacturing of a Root‐Inspired Microneedle Array Patch (R‐MAP)

The fabrication process of R‐MAP integrates 3D printing with solution‐based molding (Figure 1c). The R‐MAP consists of microneedles with a lattice‐structured base designed to mimic the intersecting mechanism of the heart‐root system. This lattice framework allows the soft substrate material to infiltrate the internal spaces during fabrication, forming a robust interlocking interface that enhances mechanical coupling and preserves structural integrity under deformation. R‐MAP mold is fabricated via a high‐resolution 3D printer (microArch S130, Boston Micro Fabrication, MA, USA). The mold is then filled with a polydimethylsiloxane (PDMS) precursor, which infiltrates the lattice architecture via capillary action. Upon thermal curing, a hybrid patch is formed wherein the rigid microneedles are embedded within the soft elastomer matrix through interfacial mechanical interlocking. The patch can be easily demolded due to controlled fracture occurring along the interface between the cured lattice and the base plate of the mold.

In conventional flexible microneedle systems fabricated through surface bonding between two dissimilar materials, the interfacial connection between the microneedles and the soft substrate often fails under deformation due to mechanical mismatch. In contrast, the R‐MAP introduces a structurally integrated design in which a rigid lattice framework is mechanically interlocked with the soft substrate. Because the microneedles and the lattice sections are both composed of rigid materials, the integrated structure between the substrate and microneedles remains intact even when the substrate undergoes deformation (Figure 1d).

To systematically assess the impact of base geometry on interfacial bonding strength, we designed multiple microneedle models incorporating distinct support architectures: a flat interface without any supporting structures, vertical supports with a simple cuboid, vertical supports with curved surface topographies resembling natural root bulges, and intersecting lattice structures (Figure 1e). Notably, the intersecting lattice design demonstrated over a threefold increase in adhesion strength (3.65 ± 0.09 N cm^−1^) relative to non‐interlocking designs (0.33 ± 0.05, 0.97 ± 0.04, 1.08 ± 0.03, and 3.65 ± 0.09 N cm^−1^, respectively), confirming that interfacial interlocking plays a critical role in maximizing bonding strength between rigid microneedles and soft substrates.

Compared to previously reported hybrid microneedle systems, which often struggle to balance effective tissue penetration and long‐term conformal adhesion, the R‐MAP offers a distinctive advantage. The root‐inspired interlocking lattice enables robust mechanical bonding between the rigid microneedles and the soft elastomeric substrate, preventing delamination under deformation. As a result, R‐MAP achieves the highest modulus mismatch ratio between microneedle and substrate among comparable systems [21, 22, 23, 24, 25, 26, 27, 28, 29, 30, 31, 32, 33, 34, 35, 36, 37, 38, 39, 40, 41, 42, 43, 44, 45], facilitating reliable skin penetration (Figure 1f). These results are further supported by a comprehensive comparison of previously reported flexible and stretchable microneedle patches composed of heterogeneous materials, including their material compositions, mechanical properties, and modulus mismatch ratios, as summarized in Table S1.

### 2.2 Mechanical Optimization of R‐MAP Design

**FIGURE 2:**
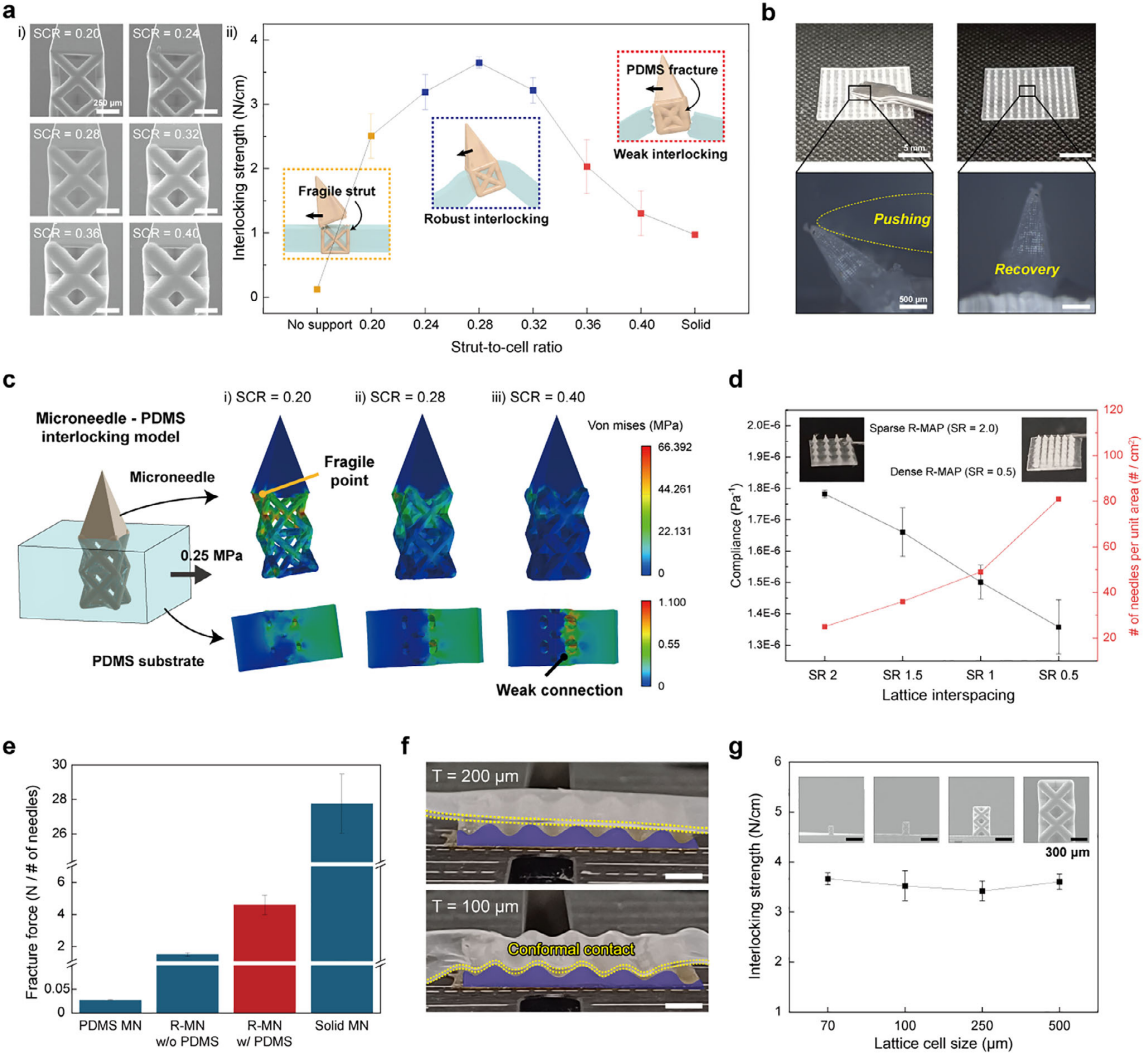
(a) (i) SEM images of root‐inspired microneedles (R‐MN) varying SCR values (Scale bar = 500 µm) and (ii) interlocking strength of root‐inspired microneedles with varying SCR values. (b) Optical images of R‐MAP forced with tweezers and optical microscopic images of individual microneedles during compression. (c) FEM‐based von Mises stress distribution in the bonded microneedle and PDMS substrate structure under shear loading, as a function of SCR. Stress contours were evaluated for three SCR conditions (SCR = 0.20, 0.28, and 0.40). Stress distribution within the R‐MN only (PDMS substrate hidden for clarity; analysis performed on the full bonded structure) and stress distribution across the entire assembly, including both the R‐MN and the PDMS substrate, are represented. (d) Trade‐off relation of mechanical compliance of R‐MAP by varying spacing ratio. (e) Fracture force per needle of various microneedles. (f) Optical images of contact conformity of stretchable substrates on dynamic surface (Scale bar = 500 µm). (g) Peel‐off adhesion profiles of thin/thick substrate (*n* = 3).

To identify an optimal microneedle base architecture that ensures both strong interfacial bonding and mechanical durability, we systematically varied the lattice geometry by modulating the Strut‐to‐Cell Ratio (SCR). This was achieved by fixing the unit cell size at 500 µm and incrementally increasing the strut thickness from 100 to 200 µm in 20 µm steps, resulting in SCR values ranging from 0.20 to 0.40 (Figure 2a (i)). We first quantified the interlocking strength between the microneedle base and the soft PDMS substrate as a function of SCR through peel‐off experiments, with the experimental setup detailed in Figure S2a. As shown in Figure 2a(ii), interfacial bonding between the lattice and the substrate was optimal within the SCR range 0.24–0.32, with the interlocking strength reaching a maximum of 3.66 N cm^−1^ at SCR = 0.32. However, at lower SCR values (≤ 0.20), the strut thickness was too thin to provide sufficient mechanical support at the junction between the lattice and the microneedle base, leading to structural failure under applied external loads. Conversely, at higher SCR values (≥ 0.36), the interstitial space became too restricted for sufficient precursor penetration, leading to incomplete filling and weak structural integration between the PDMS and the lattice structure. As a result, lattices that were not properly interlocked were pulled out during peeling due to insufficient mechanical anchoring. The raw peel‐off force data across all tested SCR conditions, along with representative images capturing the three distinct failure modes observed across the SCR range, as described above, are presented in Figure S2b,c. These results highlight that the enhanced performance of the lattice base cannot be attributed merely to a superficial increase in interfacial roughness or physical interlocking. Instead, the lattice introduces a fundamental structural mechanism in which crack propagation, regardless of direction, is continuously redirected and transformed into constrictive stresses by the surrounding struts. This multidirectional stress redistribution enables robust integration between rigid microneedles and compliant PDMS substrates. Unlike non‐intersecting root‐inspired anchoring or chemical adhesives, which are limited to specific interfacial bonding modes, the lattice architecture provides a universal geometric mechanism capable of sustaining interfacial stability against detachment in all loading directions.

The dependence of adhesion strength on the SCR further illustrates how this principle manifests in practice. The intermediate SCR regime did not simply represent an empirical optimum but corresponded to the equilibrium between two competing failure pathways: strut fracture at low SCR values and rupture of infiltrated PDMS bridges at high SCR values. Within this window, frictional interactions at the lattice–PDMS interface and the load‐bearing capacity of the PDMS bridges coexisted in balance, thereby yielding maximized adhesion. Thus, the optimized performance of the R‐MAP reflects the intrinsic mechanics of the three‐dimensional lattice, which simultaneously enforces multidirectional constriction and tunes the distribution of stresses across the heterogeneous interface. Based on the interlocking performance results, we narrowed the SCR design window to the range of 0.24–0.32, where PDMS infiltration and peel‐off strength were maximized. The R‐MAP with optimized lattice exhibited excellent mechanical resilience, withstanding bending deformation beyond 45° under external loading and fully recovering its original shape without structural damage (Figure 2b).

To further elucidate the mechanical basis of the SCR‐dependent interlocking behavior, finite element modeling was performed for representative lattice geometries with SCR values of 0.20, 0.28, and 0.40 (Figure 2c). To mimic the mechanical disturbance experienced by the patch after microneedle penetration into tissue, a shear load was applied to the PDMS substrate in the rigid microneedle–soft substrate integrated structure. At SCR = 0.20, pronounced stress concentrations emerged at the junction between the upper lattice struts and the microneedle base, as well as at the lattice crossing sites, indicating mechanically vulnerable regions that could initiate structural damage under external stimulation after insertion. In contrast, at SCR = 0.40, the thicker struts alleviated stress concentration within the lattice‐supported microneedle region itself, but substantial stress was instead concentrated in the PDMS interconnection regions confined between adjacent lattice elements, suggesting increased susceptibility of the infiltrated soft bridges to failure. Notably, at SCR = 0.28, the applied stress was distributed more evenly across both the lattice framework and the PDMS interconnection regions, indicating a balanced load‐sharing state at the heterogeneous interface. These findings support the experimental results by showing that the optimized SCR does not merely maximize interlocking geometrically, but also minimizes localized failure by redistributing external loads across both the rigid lattice and the compliant PDMS phase.

To further assess whether this lattice‐mediated interlocking strategy is specific to PDMS or can be generalized to other soft substrates, we additionally fabricated R‐MAPs using representative silicone‐based elastomers with different mechanical characteristics, including Dragon Skin and Mold Star (Figure S3). Without the lattice‐mediated interlocking structure, all elastomeric substrates exhibited only weak interfacial adhesion, with peel‐off strengths of 0.12 ± 0.02 N cm^−1^ for PDMS, 0.25 ± 0.04 N cm^−1^ for Dragon Skin, and 0.15 ± 0.04 N cm^−1^ for Mold Star. In contrast, the incorporation of the 3D‐printed lattice markedly increased the interfacial strength to 3.65 ± 0.09, 3.23 ± 0.15, and 2.86 ± 0.22 N cm^−1^ for PDMS, Dragon Skin, and Mold Star, respectively. This corresponds to approximately 29.3‐, 12.8‐, and 19.0‐fold enhancements compared with the corresponding non‐interlocking controls. These results demonstrate that the proposed mechanical interlocking mechanism is not restricted to PDMS, but can be extended to other silicone‐based soft polymeric substrates, supporting the broader material applicability of the R‐MAP design.

Furthermore, the flexibility of the 3D printing process enabled precise control over the microneedle array spacing in the R‐MAP design (Figure 2d). By modifying the 3D model of the R‐MAP mold, we fabricated patches with spacing ratios (SR) of 0.5, 1.0, 1.5, and 2.0. For each SR condition, tensile tests were performed to assess stretchability, and bending tests were conducted to evaluate flexibility (Figure S4). Lower SR values resulted in a higher microneedle density per unit area, which could enhance tissue penetration and drug delivery efficiency; however, this came at the expense of reduced compliance—namely, lower stretchability. This trade‐off was quantitatively assessed across all SR configurations, and an SR of 1.5 was selected as the final design offering an optimal balance between microneedle density and mechanical compliance. The R‐MAP design optimized for both SCR and SR exhibited robust mechanical durability under repeated deformation. Specifically, it demonstrated high stretchability up to 75% strain and withstood multidirectional torsion without structural failure (Figure S5). In addition, the patch maintained its mechanical performance without structural failure after 100 cycles of stretching and relaxation at 50% strain (Figure S6).

To assess the structural integrity of the final R‐MAP design during insertion, we evaluated the fracture force under four different conditions: MAP made entirely of PDMS (PDMS‐MAP), R‐MAP without interlocked PDMS, R‐MAP with interlocked PDMS substrate, and 3D‐printed Solid MAP (Figure 2e). The result shows that PDMS‐MAP exhibited a fracture force of less than 0.05 N per needle, which was insufficient to ensure reliable tissue penetration. However, R‐MAP without PDMS yielded fracture forces well above the minimum required force for skin penetration (0.1 N per needle), confirming their functional applicability. Furthermore, the R‐MAP with interlocked PDMS exhibited a 204% increasing of fracture force (4.60 ± 0.61 N) relative to R‐MAP without PDMS (1.51 ± 0.12 N), highlighting the critical role of the interlocked soft substrate in reinforcing mechanical stability. These results indicate that this configuration achieves approximately half the stiffness of Solid MAP, which can be attributed to the combined effects of load distribution within the lattice architecture itself and additional load dissipation through its integration with the PDMS substrate. The superior performance of R‐MAP is attributed to an optimal trade‐off between sufficient interstitial volume for precursor infiltration and a sufficiently dense lattice structure for mechanical anchoring of the microneedle base.

In curved anatomical regions such as the oral mucosa, not only material flexibility but also patch thickness plays a decisive role in achieving conformal contact. As shown in Figure 2f, patches with a thickness of approximately 200 µm failed to conform to the tissue curvature, whereas those thinned to 100 µm readily adapted to the curved surfaces. This improved conformal contact not only facilitated uniform microneedle engagement across the tissue surface but also significantly enhanced the adhesive stability of the patch. To enable such thickness tuning of the R‐MAP, it was essential that the lattice structure maintain its interlocking functionality across various size scales. To evaluate this, we fabricated arrays of lattices with unit cell sizes ranging from 500 µm down to 70 µm and tested their interlocking performance with PDMS substrates (Figure 2g and Figure S7). Notably, effective interlocking was consistently retained even in the lattice with a 70 µm unit cell size. Based on these results, we fabricated a thin R‐MAP design with a total thickness below 100 µm, which enabled conformal adaptation to complex biological surfaces.

### 2.3 Functionalization of R‐MAP With Mucoadhesive Layer of Sustained Tissue Adhesion

**FIGURE 3:**
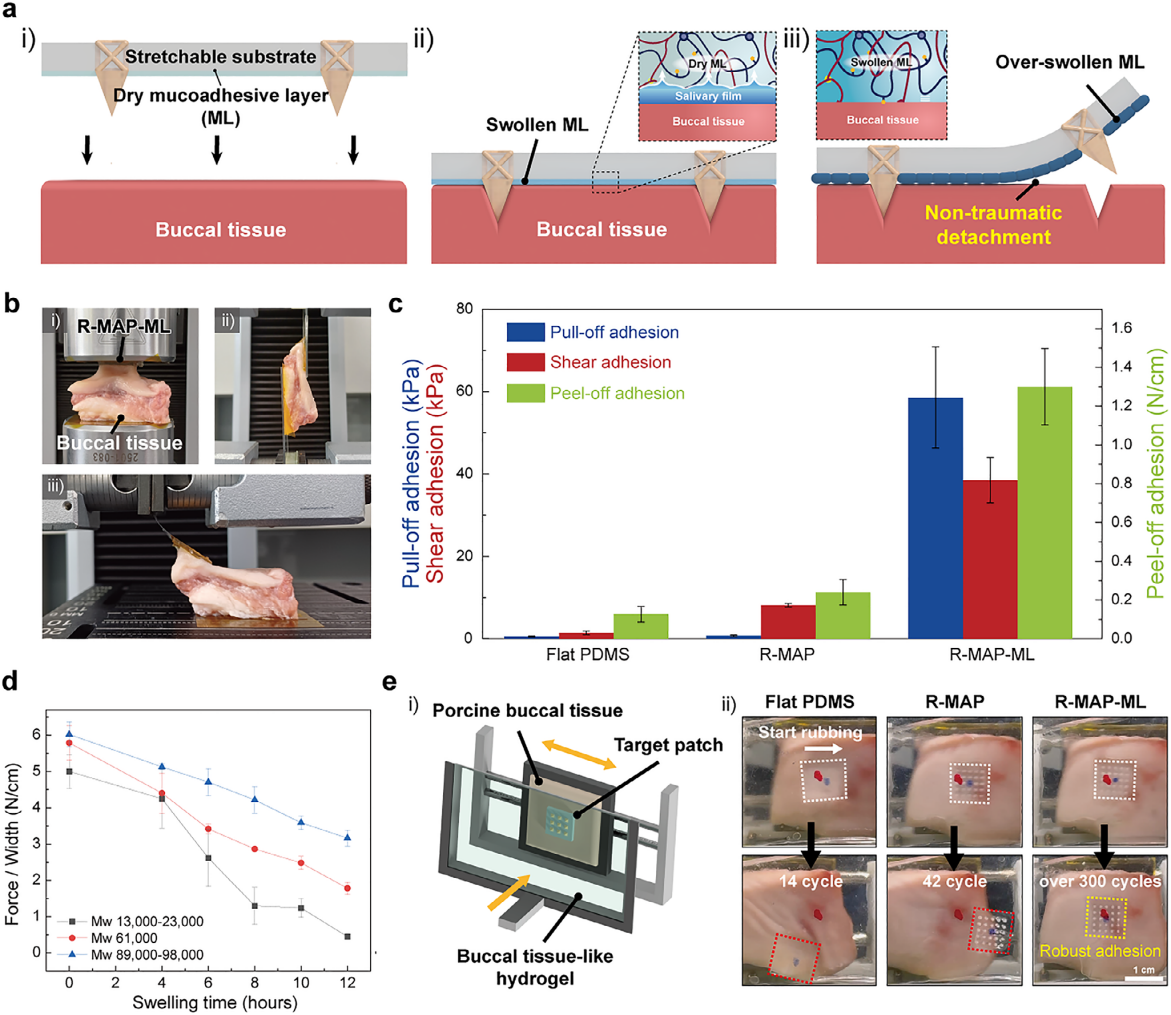
(a) Schematic of the adhesion process of the mucoadhesive layer. (b) Adhesion performances of R‐MAP‐ML on (i) pull‐off, (ii) shear, and (iii) peel‐off direction. (c) Profile of adhesion strength by swelling time of the mucoadhesive layer. (d) Swelling time‐dependent peeling adhesion forces of the mucoadhesive layer consist of PVAs with different molecular weights) (e) (i) Schematic of 1‐axis linear friction actuator and (ii) optical images of ex vivo friction test.

To enhance the intrinsic adhesion of the R‐MAP to wet biological surfaces, we integrated a mucoadhesive layer (ML) onto the patch surface (Figure 3a). The hydrogel coating facilitates robust adhesion on wet mucosal surfaces by rapidly displacing interfacial water through swelling‐induced absorption. This is followed by the formation of multiple intermolecular interactions, including hydrogen bonding, electrostatic attraction, and, where applicable, covalent crosslinking with tissue amine groups that collectively establish stable and durable tissue bonding (Figure S8) [46]. As the hydrogel progressively absorbs water from the surrounding environment over time, its adhesion strength gradually decreases, eventually allowing for non‐traumatic detachment of the patch without causing tissue irritation [47]. We selected polyvinyl alcohol (PVA) with a specific molecular weight to precisely tune the swelling ratio of the mucoadhesive layer and to control the time required to reach its over‐swollen state. The coating process involved surface activation of the R‐MAP via benzophenone‐mediated photochemical treatment, promoting hydrophilic modification and covalent crosslinking with the hydrogel network [48, 49]. Subsequently, the hydrogel precursor solution was applied to the activated surface, followed by UV curing to form the mucoadhesive layer (Figure S9) [50, 51]. Successful formation of the ML was further confirmed by Fourier transform infrared (FT–IR) spectroscopy, which exhibited characteristic absorption bands at 1158 and 1209 cm^−1^ assigned to C─N─C stretching of the NHS ester, together with a prominent band at 1696 cm^−1^ corresponding to the carboxylic acid C═O stretching of PAA, verifying the successful incorporation of the mucoadhesive chemistry onto the R‐MAP surface (Figure S10). This process ensured uniform hydrogel deposition while preserving the structural integrity and mechanical properties of the underlying microneedle architecture [52, 53].

The adhesion performance of the R‐MAP‐ML was quantitatively evaluated using ex vivo porcine buccal tissue, simulating clinically relevant mucosal environments (Figure 3b and Figure S11). In the oral cavity, patches are subjected to multi‐directional forces, necessitating robust adhesion in various detachment modes. To evaluate this, we measured adhesion strength under three distinct loading conditions, including pull‐off, shear, and peel‐off (Figure 3c). Baseline measurements using flat PDMS substrates yielded negligible adhesion, with pull‐off (0.56 ± 0.11 kPa) and shear strengths (1.44 ± 0.45 kPa) underscoring the inherent difficulty of achieving wet‐tissue adhesion. While the introduction of R‐MAP architecture without the mucoadhesive layer provided a modest increase in shear strength (8.17 ± 0.42 kPa) due to the mechanical anchoring of the microneedles, its pull‐off strength remained low (0.75 ± 0.16 kPa), indicating insufficient interfacial affinity. In striking contrast, the R‐MAP‐ML exhibited a robust adhesion profile, achieving a pull‐off strength (58.6 ± 12.3 kPa) and a shear strength (38.5 ± 5.5 kPa) representing remarkable 64.6‐ and 4.8‐fold enhancements over the uncoated R‐MAP. Further characterization via peel‐off tests demonstrated a high interfacial toughness of 1.3 ± 0.2 N cm^−1^, confirming that the mucoadhesive layer effectively bridges the gap between mechanical interlocking and chemical bonding. These results confirm the strong affinity of the mucoadhesive interface under hydrated conditions typical of the oral cavity.

In addition to robust initial adhesion, controlled detachment is critical for clinical applications to minimize tissue irritation during removal. As the mucoadhesive layer progressively absorbs moisture over time, its adhesive strength gradually decreases. Notably, the swelling ratio of the ML, and consequently its adhesion strength and retention duration, varies depending on the molecular weight of the PVA used. To identify a formulation that ensures sufficient reduction in adhesion robustness at the intended detachment time point, we systematically tested MLs composed of PVA with molecular weights (Mw) of 13 000, 61 000, 89 000, and 145 000. Each patch was applied to tissue and subjected to continuous hydration under underwater conditions, while the peel‐off adhesion was measured at various swelling time points. Among these, the ML fabricated with 13 000 MW PVA showed a retention of 52.4% of its initial peel‐off strength after 6 h of hydration, and a reduction to below 10% after 12 h (Figure 3d). This hydration‐responsive weakening enables gentle and non‐traumatic removal of the patch following prolonged application.

To further evaluate the ability of the R‐MAP‐ML to resist mechanical stimuli resembling intraoral friction, we developed a custom‐built uniaxial linear actuator to simulate tissue movement (Figure 3e and Figure S12). For comparative analysis, three patch types (flat PDMS, R‐MAP, and R‐MAP‐ML) were tested. Each patch was applied to ex vivo porcine oral tissues, and the opposing surface was modeled using a transparent hydrogel designed to replicate the viscoelastic properties of oral mucosa. The two surfaces were compressed under a normal load of 30 g cm^−2^, which is the physiological pressure between the buccal mucosa and gingiva [54], while reciprocating lateral sliding motion was applied to mimic intraoral friction. The flat PDMS patch detached rapidly, losing adhesion entirely within 14 cycles. The R‐MAP, benefiting from microneedle‐mediated anchoring within the tissue, exhibited improved retention but was fully displaced after 42 cycles. In contrast, the R‐MAP‐ML maintained complete attachment for over 300 cycles under identical test conditions, indicating a substantial enhancement in adhesion robustness due to the synergistic contribution of microneedle interlocking and mucoadhesive coating (Movie S1).

### 2.4 In Vivo Assessment of R‐MAP Adhesion

**FIGURE 4:**
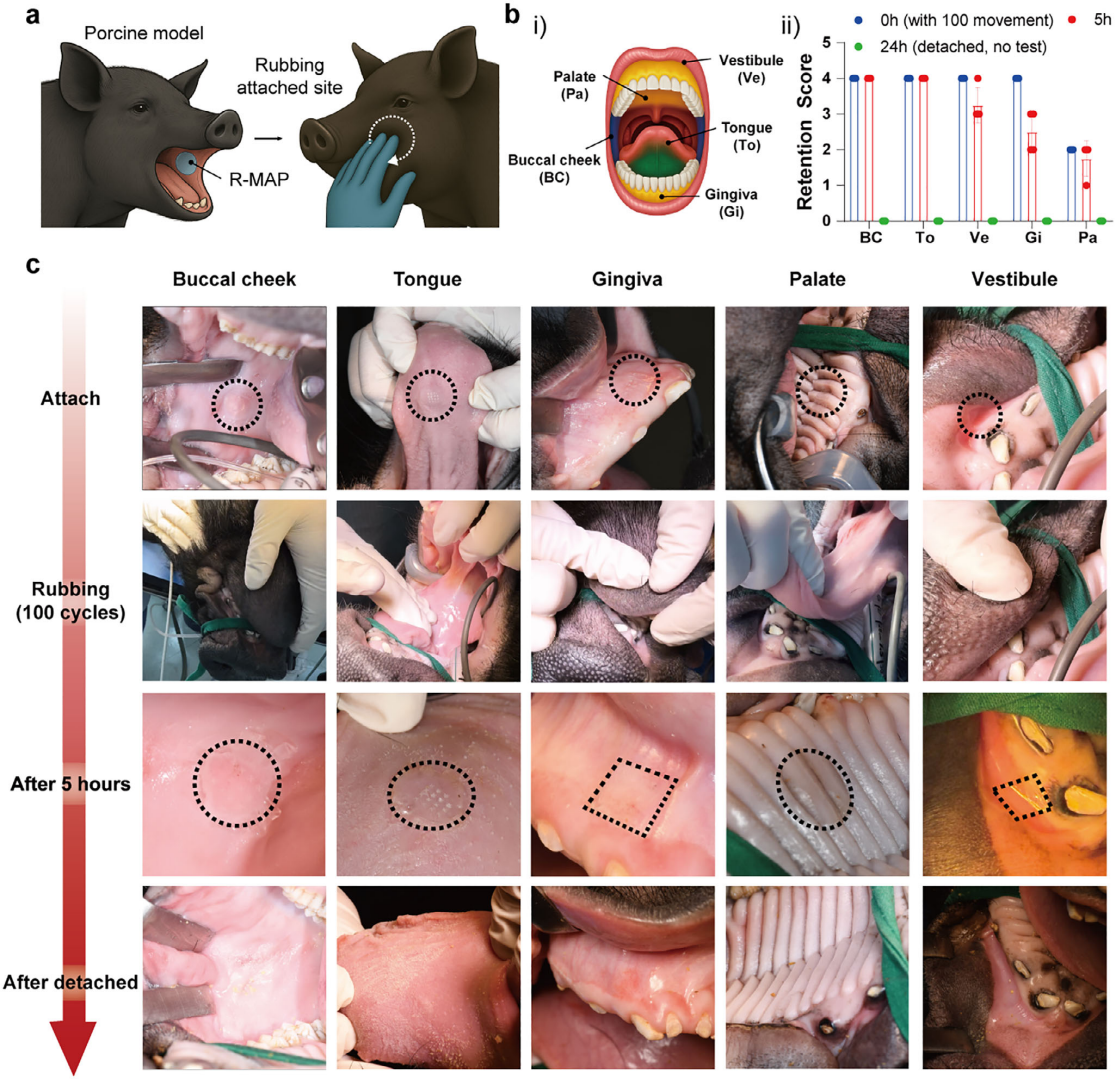
(a) Schematic illustration of in vivo assessment process using laboratory pig. (b) (i) Anatomical illustration of target tissue and (ii) retention score (*n* = 4). (c) Representative images of R‐MAP attachment at different time points across each application site during the in vivo adhesion assessment.

To evaluate the adhesive performance of R‐MAP‐ML under in vivo conditions, 100 manual friction stimulations were applied to simulate natural oral movements (Figure 4a). Patches were tested across five intraoral anatomical regions (Figure 4b(i)) in a miniature‐pig model. Notably, the oral environment of the miniature pig naturally provided abundant saliva, and the physiological body temperature was strictly maintained under anesthesia, closely mimicking realistic dynamic intraoral conditions. Patch adhesion was evaluated immediately after application following 100 manual friction stimulations, after an additional 100 friction stimulations at 5 h post‐application, and at 24 h post‐application. No mechanical testing was performed at 24 h because all patches had completely detached. The entire experimental procedure is illustrated in detail in Figure S13 and Movie S2, and retention scores are summarized in Figure 4b(ii), and the criteria used to assign the retention scores are provided in Table 1. At 0 h, the buccal cheek (BC), tongue (To), and vestibule (Ve) retained the patches completely (4.0 ± 0.0). Attached gingiva (Gi) showed slightly lower adhesion (3.5 ± 0.5), whereas the palate (Pa) displayed the poorest initial retention (2.0 ± 0.0). After 5 h, the BC and To sites still exhibited full retention (4.0 ± 0.0). Retention declined modestly at Ve (3.0 ± 0.0) and Gi (2.5 ± 0.5) and fell further at Pa (1.5 ± 0.5). By 24 h, all patches were completely detached at every site (0.0 ± 0.0), precluding mechanical testing at that time‐point. The R‐MAP‐ML remained securely attached for over 5 h_s on most movable mucosa (BC, To, Ve), but adhesion was weaker on keratinized or contoured tissues such as attached gingiva and palate. Because all patches detached within 24 h, the clinically relevant performance window appears to be the first few hours after application.

**TABLE 1:** Definition of retention scores used to evaluate the degree of patch adhesion on the mucosal surface.

Score	Definition
4	Fully adhered across the entire surface, with no visible lifting or disruption
3	Mostly adhered with minor edge lifting or localized detachment (< 25% of the area)
2	Partial adhesion maintained; moderate detachment visible (25%–50% of the area)
1	Poor adhesion; most of the patch detached, only small area remains attached (> 50% detached)
0	Completely detached with no visible adhesion on the mucosa

To confirm that the R‐MAP achieved effective tissue engagement during the adhesion study, H&E‐stained cross‐sections of porcine oral mucosa were examined. Clearly defined microneedle insertion tracks extending into the connective tissue were observed, with measured penetration depths of 691–703 µm (∼70% of the 1000 µm needle height), confirming reliable mechanical anchorage throughout the retention period (Figure S14).

The R‐MAP‐ML demonstrated robust muco‐adhesion and excellent biocompatibility across a uniquely wide spectrum of intra‐oral sites. Applied to the buccal cheek, tongue, vestibule, attached gingiva, and hard palate in a miniature‐pig model, the patch remained securely in place for over 5 h in most regions (retention score 4.0 ± 0.0 even on the highly mobile buccal and lingual mucosae). All patches then released spontaneously by 24 h without any signs of tissue trauma (Figure 4c). This timeline shows the patch fulfils its intended short‐term role and then detaches gently—an outcome that is desirable for practical use. Notably, to our knowledge this is the first study to evaluate a single muco‐adhesive system on such diverse, anatomically and mechanically distinct intra‐oral surfaces in the same animal. The oral cavity is not a simple flat plane: it contains highly keratinized, rigid areas (hard palate, attached gingiva), soft and constantly mobile tissues (tongue, buccal mucosa), and sharply curved, moisture‐retaining recesses (vestibule) interspersed with hard‐tissue obstacles (teeth). Demonstrating reliable adhesion under this breadth of topological and mechanical challenges adds a layer of clinical realism that has been largely missing from previous reports focused on a single site. It is worth noting that determining the exact maximum retention time in vivo is constrained by veterinary guidelines, which limit the frequency and duration of general anesthesia required for intraoral inspections. Furthermore, the natural behaviors of the miniature pig—such as continuous mastication and hypersalivation—create an exceptionally harsh baseline environment. Under more stable, non‐extreme ex vivo conditions, the patches demonstrated the capacity to maintain functional adhesion for up to 12 h. Therefore, in human applications with standard mucosal movement, the effective retention time is expected to extend well beyond the 5 h confirmed here, providing a sufficient therapeutic window before programmed detachment.

### 2.5 Drug Delivery and Histological Analysis

To evaluate whether the R‐MAP can actively deliver compounds through the penetration pathways confirmed above, proof‐of‐concept studies were performed using both an in vitro agarose gel model and a murine skin model. Rat dorsal skin was selected as a generalizable model for evaluating compound delivery through the microneedle–mucoadhesive interface. Sulforhodamine B (MW: 479.0 Da), a fluorescent model compound, was incorporated into the mucoadhesive layer during fabrication. For the in vitro dish‐based assessment, the dye‐loaded R‐MAP‐ML was applied to agarose gel as a tissue‐mimicking substrate, where brightfield imaging confirmed microneedle penetration and fluorescence imaging revealed localized sulforhodamine B distribution along the penetrated region, indicating model compound release from the mucoadhesive layer after insertion (Figure S15). The dye‐loaded R‐MAP‐ML was then applied to the dorsal skin of male Sprague–Dawley rats (*n* = 3) for 12 h. Fluorescence imaging of tissue cross‐sections revealed concentrated sulforhodamine B signals at discrete sites corresponding to microneedle insertion points, while no fluorescence was detected in control tissue (Figure S16). Overlay of brightfield and fluorescence channels confirmed spatial colocalization of dye accumulation with regions of epithelial disruption consistent with microneedle penetration tracks. This distribution is consistent with the delivery mechanism of the R‐MAP, as the non‐biodegradable microneedles are not designed to release compounds through needle dissolution; instead, compound release is mediated by the hydrated mucoadhesive layer at the tissue–needle interface.

To quantitatively assess the release of the model compound over time, ex vivo permeation studies were performed using a vertical Franz diffusion cell over application durations ranging from 0 to 72 h. Dye‐loaded R‐MAP‐ML released 29.9 ± 9.1%, 45.0 ± 9.7%, 60.1 ± 6.9%, 66.6 ± 6.4%, 77.6 ± 9.8%, 80.9 ± 7.2%, and 83.4 ± 5.9% of the total loaded sulforhodamine B at 2, 4, 8, 12, 24, 48, and 72 h, respectively, indicating rapid initial release that gradually approached a plateau by 24–48 h (Figure S17). In contrast, dye‐free R‐MAP‐ML exhibited only background‐level signal (≤ 0.8% at all time points), confirming that the detected fluorescence originated specifically from the loaded compound rather than from the patch material itself. These results confirm that the R‐MAP‐ML is capable of actively delivering the model compound in a time‐dependent manner, consistent with the spatial distribution observed in the fluorescence imaging above.

**FIGURE 5:**
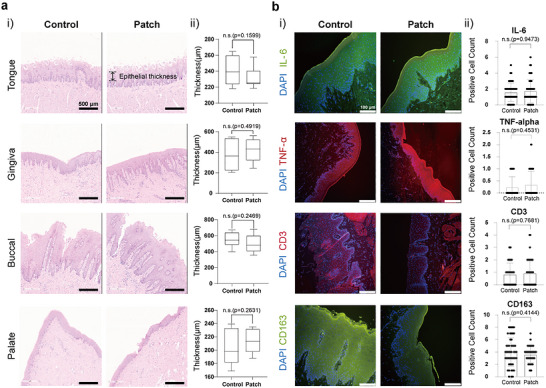
(a) (i) Representative H&E sections of tongue, attached gingiva, buccal cheek and palate 24 h after patch removal. Patch‐treated mucosa shows a continuous epithelial surface and absence of overt inflammatory‐cell infiltration, matching the corresponding control tissue (scale bar = 100 µm). (ii) epithelial‐thickness measurements for each site (12 measurements per group; two animals) plotted as median ± interquartile range with 1.5 × IQR whiskers. No significant difference was detected between control and patch groups at any site (*p* > 0.15, unpaired two‐tailed t‐test; n.s.). (b) (i) Merged fluorescence images from four anatomical sites: IL‐6 (palate), TNF‐α (palate), CD3 (dorsal tongue), CD163 (attached gingiva) and Pan‐cytokeratin (buccal mucosa). Target signals appear in green (IL‐6, CD163) or red (TNF‐α, CD3); nuclei are counter‐stained with DAPI (blue). Scale bars = 50 µm. (ii) Positive cell counts for each marker (50 ROIs of 0.052 mm^2^ per group). Boxes represent the 25th–75th percentiles, center lines the median, and whiskers extend to 1.5 × IQR; individual ROI values are plotted as dots. Mann–Whitney tests yielded non‐significant p values for IL‐6 (0.9473), TNF‐α (0.4531), CD3 (0.7681) and CD163 (0.4144), indicating no measurable difference between control and patch samples for the parameters assessed.

Furthermore, to evaluate the safety and biocompatibility of the mucoadhesive layer during detachment, histological examination was performed on the miniature‐pig oral mucosa described in Section 2.4, following spontaneous patch detachment at 24 h. The results revealed well‐preserved epithelial integrity across all tested anatomical sites. No significant tissue damage, such as surface erosion or epithelial detachment, was observed in the majority of samples, confirming that peeling off the strongly adhered patch does not induce mechanical trauma to the mucosal surface. The palate showed the best overall preservation, consistent with its naturally thick, keratinized epithelium. The vestibular mucosa displayed occasional, shallow surface irregularities in a minority of sections, but these were focal and not accompanied by inflammatory cell influx or tissue necrosis. The H&E findings indicate that the microneedle patches detached gradually in the moist oral environment without provoking overt tissue irritation or injury. (Figure 5a (i)) These observations were further corroborated by pan‐cytokeratin immunostaining, which demonstrated continuous and uniform epithelial expression without evidence of cytokeratin loss in the buccal mucosa (Figure S18). Second, quantitative analysis revealed no significant difference in epithelial thickness between patch‐treated and control mucosa at any oral site (Figure 5a(ii)). Mean ± SD values (control vs. patch, *n* = 12 measurements per group) were: palate, 203 ± 26 µm vs. 214 ± 18 µm; buccal cheek, 553 ± 91 µm vs. 504 ± 111 µm; tongue, 242 ± 18 µm vs. 232 ± 13 µm; and attached gingiva, 375 ± 140 µm vs. 411 ± 110 µm. These findings indicate that microneedle‐patch applications do not induce epithelial thinning, hyperplasia, or other structural alterations in any of the examined intra‐oral regions. Lastly, quantitative image‐based cell counts showed no significant difference between control and patch tissues (Figure 5b). For each marker, 50 ROIs were analyzed per group, and Mann–Whitney tests yielded non‐significant p values. (IL‐6, 0.9473; TNF‐α, 0.4531; CD3, 0.7681; CD163, 0.4144).

## 3 Conclusion

This study introduces a root‐inspired microneedle array patch (R‐MAP) that leverages an architected lattice design to resolve the long‐standing challenge of integrating rigid microneedles with soft, curved, and hydrated biological tissues. Unlike conventional planar or chemically bonded configurations, which often suffer from delamination or mechanical mismatch under deformation, the R‐MAP utilizes a geometrically interlocked interface that combines mechanical anchoring with conformal surface adaptation. By tuning the strut‐to‐cell ratio (SCR) and spacing ratio (SR) of the lattice base, we demonstrated that mechanical durability and interfacial adhesion can be independently optimized. The R‐MAP showed high fracture strength and reversible bending up to 45°, confirming its resilience under mechanical stress. Moreover, its ultrathin form factor (below 100 µm) allowed tight conformity to highly curved anatomical sites, such as the inner oral mucosa, where conventional thick patches fail to adhere evenly. This thickness tunability, enabled by scalable high‐resolution 3D printing, ensures compatibility across a range of target tissue geometries.

To further improve adhesion on wet mucosal surfaces, we integrated a mucoadhesive layer (ML) via benzophenone‐mediated photografting. The resulting R‐MAP‐ML achieved robust initial adhesion through synergistic effects of microneedle penetration and hydration‐driven interfacial bonding. The mucoadhesive layer also enabled time‐dependent weakening of adhesion via controlled swelling, facilitating non‐traumatic removal, which is a key clinical requirement. Because prolonged mechanical anchoring in the oral cavity can lead to tissue maceration and hygiene issues, this hydration‐responsive detachment within 24 h serves as a built‐in safety feature tailored for short‐to‐medium‐term mucosal applications. In dynamic friction simulations mimicking intraoral conditions, the R‐MAP‐ML maintained secure attachment over 300 cycles of shear, significantly outperforming non‐adhesive controls.

Compared to previously reported microneedle systems, the R‐MAP platform distinguishes itself through a unified design strategy that balances penetration strength, conformability, adhesion, and mechanical resilience, all achieved without complex chemical surface modifications or multilayer laminations. In vivo evaluation in a miniature‐pig model confirmed that the R‐MAP‐ML maintained secure mucosal adhesion for over 5 h across multiple intraoral sites without histological evidence of tissue damage, demonstrating both functional reliability and biocompatibility under physiologically relevant conditions. Future work will focus on long‐term therapeutic efficacy studies, pharmacokinetic validation of transmucosal drug delivery, and clinical translation to human subjects. In the long term, the modular design of the R‐MAP architecture, which decouples the rigid and soft interface elements, may be applied to a wide range of biointerfaces beyond microneedle delivery, including stretchable electronics, implantable biosensors, and dynamically adaptive wound care systems. Furthermore, the compatibility of this design with high‐resolution additive manufacturing suggests strong potential for scalable clinical translation across multiple therapeutic areas, particularly those requiring robust yet reversible adhesion to soft, wet, and dynamic tissues.

## 4 Experimental Section/Methods

### 4.1 Biological Models and Approval

All animal work complied with the Guide for the Care and Use of Laboratory Animals (eighth ed., NRC 2011) and the Korean Laboratory Animal Act, and reporting follows ARRIVE 2.0 guidelines. The Institutional Animal Care and Use Committee of the Pre‐clinical Research Resources Center, Samsung Medical Center (Seoul, Republic of Korea) approved the protocol (IACUC #20240805001, 5 Aug 2024) and the Institutional Animal Care and Use Committee of Kyung Hee University (Approval No. KHSASP‐25‐351). Two female Yucatan miniature pigs (Kronex, Cheongju, South Korea; RRID:NSRRC_0012; ∼80 kg body weight) were used for the in vivo intraoral adhesion and histological evaluations.

### 4.2 Root‐Inspired Microneedle Array Patch (R‐MAP) Design and Modeling

R‐MAP is composed of two main components: (1) a rigid microneedle (MN) array where each MN is mounted on a lattice base, which serves as the mold, and (2) a flexible PDMS layer that fills the lattice base structure and cures to form a mechanically interlocked interface. The rigid MN‐lattice mold structure was designed using nTop (NY, USA), a software specialized in generating complex lattice geometries. The MNs were modeled with a base width of 500 µm and a height of 1000 µm, where the base of each MN incorporated a two‐layer lattice structure with unit cell sizes of 70, 100, 250, and 500 µm. Strut thicknesses were scaled proportionally to the strut‐to‐cell size ratio defined in the 500 µm model, which had a strut thickness of 140 µm. All MNs were arranged in a 5 × 5 two‐dimensional array with a center‐to‐center needle spacing of 1.5 times the unit cell size.

### 4.3 High Resolution 3D Printing of R‐MAP Molds

R‐MAP molds were fabricated using a high‐resolution projection microstereolithography (PµSL) 3D printer, microArch S130 (BMF, USA), with BMF BIO Resin (BMF, USA). The molds were then carefully detached from the build platform using a scraper and rinsed thoroughly with fresh 99% isopropyl alcohol (IPA;Daejung, South Korea) to remove uncured resin. The molds were then placed on a clean Petri dish and post‐cured using a FormCure (Formlabs, USA) for 2 h at 50°C under 405 nm light. Following the post‐curing, the molds underwent secondary washing by immersion in a fresh 99% IPA bath to remove the residual photoinitiator. After soaking, the molds were rinsed again with fresh IPA and air‐dried at room temperature for at least 2 h. The geometry of the molds was visually inspected using scanning electron microscope (SEM) imaging using a JSM‐IT800 (JEOL, Japan) at the Cooperative Center for Research Facilities (CCRF), Sungkyunkwan University.

### 4.4 Fabrication of R‐MAP

A prepolymer solution of polydimethylsiloxane (PDMS; Sylgard 184, Dow, USA) was prepared by mixing the base and curing agent at a 10:1 weight ratio. To fabricate a soft PDMS substrate, undercured PDMS was used by exploiting the curing‐inhibition effect of the resin mold, which reduced the modulus of the substrate [55]. Approximately 0.5 g of the PDMS mixture was cast onto the mold. To facilitate thorough penetration of the PDMS into the microstructured lattice features and eliminate trapped air bubbles, the mold was placed in a vacuum chamber for 1 h. The PDMS was then thermally cured at 80°C for 2 h. After cooling, the R‐MAP was demolded and stored in a desiccator until further use.

### 4.5 Fabrication of Mucoadhesive Layer (ML)

A mucoadhesive hydrogel precursor solution, designated as double‐sided tape (DST), was prepared by dissolving 35 wt.% acrylic acid, 7 wt.% poly(vinyl alcohol), 0.2 wt.% α‐ketoglutaric acid, 0.05 wt.% poly(ethylene glycol) dimethacrylate (PEGDMA), and 3 wt.% acrylic acid N‐hydroxysuccinimide (AAc‐NHS) ester in 54.75 wt.% deionized water, yielding a total weight of 10 g. All components were thoroughly mixed using a vortex mixer until fully dissolved. The surface of the previously fabricated R‐MAP was treated with an oxygen plasma system PS‐150 (Plasol, South Korea) for 5 min to improve surface hydrophilicity. The plasma‐activated R‐MAP was then immersed in a benzophenone solution for 20 min, followed by rinsing with IPA to remove excess photoinitiator. Subsequently, the DST solution was evenly applied onto the treated R‐MAP surface and cured under UV light for 30 min. To incorporate a model fluorescent compound into the hydrogel matrix, a Rhodamine B solution (5 µg/mL in deionized water) was applied onto the surface of the UV‐cured hydrogel at a volume of 2 mL/cm^2^, corresponding to a total loading of 10 µg of Rhodamine B per cm^2^ of patch area. The Rhodamine B‐loaded hydrogel was then dried for 12 h under vacuum, protected from UV light, during which the fluorescent solution was absorbed into the hydrogel network and simultaneously dried. The resulting R‐MAP‐ML was completely dried in a vacuum desiccator and sealed in airtight plastic bags containing silica gel desiccant. Final products were stored at −20°C prior to use.

### 4.6 Mechanical Characterization of Interlocking Strength

To evaluate the interfacial bonding strength between the 3D‐printed lattice array and the PDMS substrate, mechanically interlocked specimens were fabricated and subjected to a 90° peel test using a universal testing machine 34SC‐1 (Instron, USA) equipped with a 1 kN load cell at a constant crosshead speed of 30 mm/min. To systematically evaluate the effect of Strut‐to‐Cell Ratio (SCR) on interlocking strength, lattice arrays with a fixed unit cell size of 500 µm were fabricated with strut thicknesses incrementally varied from 100 to 200 µm in 20 µm steps, yielding SCR values ranging from 0.20 to 0.40. Each lattice array was densely arranged within a 44 mm × 26 mm area on a 1 mm‐thick base plate and 3D printed. The fabricated lattice arrays were then mounted onto the bottom of a custom mold, into which a PDMS prepolymer mixture (base to curing agent, 10:1 w/w) was poured following vacuum degassing. Excess PDMS was removed to match the lattice height, ensuring no overflow, and the construct was thermally cured at 80°C for 2 h to yield mechanically interlocked lattice–PDMS specimens. Peeling force–displacement curves were recorded, and interlocking strength was determined by dividing the maximum peeling force by the width of the specimen. To further assess the scalability of the interlocking performance, four additional lattice arrays with unit cell sizes of 70, 100, 250, and 500 µm were fabricated and subjected to the same peel test protocol described above.

### 4.7 Mechanical Simulation of Interlocking Strength

Finite element method (FEM) simulations were conducted using Nastran In‐CAD (Autodesk, USA) to investigate the stress distribution within the lattice‐based microneedle–PDMS interlocking structure under lateral loading. A three‐dimensional model of the integrated structure was established and automatically meshed with free tetrahedral elements. All materials were assumed to have linear elastic properties. For the simulation, a lateral stress of 0.25 N cm^−2^ was applied to the side surface of the PDMS substrate, while the microneedle region was subjected to a fixed constraint. Based on these conditions, the stress distributions in the lattice framework and the PDMS substrate were analyzed.

### 4.8 Mechanical Characterization of Microneedle Arrays

To validate the mechanical integrity, 25‐needle R‐MAP and R‐MAP mold prior to PDMS casting were subjected to compression testing using a universal testing machine 34SC‐1 (Instron, USA) equipped with a 1 kN load cell. Both sample types shared identical lattice‐based microneedle architectures, with the R‐ MAP mold lacking the PDMS substrate. Samples were mounted on a flat compression plate and force–displacement curves were recorded at a constant strain rate of 100 µm s^−1^ until the applied force reached 50 N. The fracture force of each needle was determined from the first local maximum on the force–displacement curve to compare the mechanical robustness with and without PDMS support.

### 4.9 Adhesion Strength Measurement

Adhesion strength was evaluated using three patch configurations (flat control patch, R‐MAP, and R‐MAP‐ML). Tests were conducted on fresh porcine oral mucosal tissue using a universal testing machine 34SC‐1 (Instron, USA) equipped with a 1 kN load cell. All patches were fabricated as 1 cm × 1 cm squares and tested under both dry and wet conditions. In the pull‐off test, the mucosal tissue was fixed onto the lower stage, while the patch was mounted on the upper stage. A preload of 30 kPa was applied and maintained for 10 s to ensure conformal contact between the patch and the tissue. The patch was then detached vertically at a constant rate of 3 mm s^−1^ until complete separation. In the shear test, the mucosal tissue and patch were fixed on opposing faces of a vertical testing platform. A preload of 30 kPa was applied and held for 10 s, after which the patch was sheared laterally from the tissue at a speed of 3 mm s^−1^. For wet adhesion tests, porcine oral mucosal tissues were gently rinsed and pre‐wetted with phosphate‐buffered saline (PBS) immediately prior to testing. A preload of 30 kPa was applied and held for 10 s, after which the patch was sheared laterally from the tissue at a speed of 3 mm s^−1^.

### 4.10 Ex Vivo Friction Test

To assess the frictional stability of the patch under physiologically relevant dynamic stress, an ex vivo rubbing test was performed using freshly excised porcine buccal tissue. The patch was applied onto the mucosal surface, and a repetitive lateral rubbing motion was applied against the patch using a fully swollen polyacrylamide (PAAm) hydrogel. The PAAm hydrogel was synthesized by photopolymerizing a solution containing 2 mol L^−1^ of acrylamide (AAm) as the monomer and 4 µL of 0.1 mol L^−1^ N,N'‐methylenebisacrylamide (MBAA) as the cross‐linker per 1 mL of solution. To ensure structural integrity and interfacial bonding during the test, 1.9 µL of 3‐(trimethoxysilyl)propyl methacrylate (TMSPMA) was incorporated as a coupling agent, which was selected to mimic the mechanical softness and compliance of opposing oral tissue. To simulate the environment of the oral cavity, a commercial artificial saliva (Xeromia, Hanmi Pharm) was continuously applied to the interface throughout the procedure. The experiment was conducted using a custom‐built friction testing setup. A normal compressive stress of 3 kPa was applied between the hydrogel and the tissue–patch interface, replicating the average maximal pressure exerted between the mandibular and maxillary buccal surfaces during mastication. The rubbing motion was applied in a unidirectional manner along the left–right axis. Each sample was subjected to 300 consecutive rubbing cycles under constant load. The number of cycles at which the patch fully detached from the mucosal surface was recorded.

### 4.11 In Vivo Adhesion Assessment

R‐MAP‐MLs were applied to five intra‐oral sites (buccal cheek mucosa, tongue, oral vestibule, attached gingiva and hard‐palate mucosa). For each site, two patches were placed bilaterally (one on the left and one on the right) in each animal, resulting in four replicates per site across the two animals. Before placement, the mucosa was gently dried with sterile gauze, and each patch was pressed onto the tissue for 30 s using consistent thumb pressure. An adjacent, untreated area of comparable size served as the internal control. To approximate masticatory shear forces, a board‐certified oral and maxillofacial surgeon manually delivered 100 anterior–posterior friction strokes (∼ 1 N per stroke) immediately after patch placement and again just before the 5 h assessment. Throughout the procedure, the animal's natural hypersalivation ensured a fully lubricated mucosal environment, and the core body temperature was continuously monitored and maintained within the physiological range by a veterinary team. Patch retention was evaluated at 0, 5 and 24 h with a five‐point ordinal scale.

### 4.12 In Vitro Drug Release in Agarose Gel Model

A 2% (w/v) agarose gel was prepared by dissolving agarose powder in phosphate‐buffered saline (PBS) at 90°C until fully dissolved. The solution was poured into a Petri dish and allowed to solidify at room temperature prior to use. Sulforhodamine B (SRB)‐loaded microneedle patches were applied to the surface of the solidified agarose gel and manually pressed against the gel for 30 s to ensure adequate microneedle insertion. The patches were then maintained in contact with the gel surface for 30 min at room temperature to allow sufficient diffusion of SRB into the gel matrix. Following the incubation period, the patches were carefully removed, and the agarose gel was placed on a glass slide for imaging. Fluorescence and bright‐field images were acquired using a Leica DMi8 fluorescence microscope (Leica Microsystems, Germany) equipped with an RHOD_LP filter set, with excitation at 535/50 nm and a long‐pass emission filter at 580 nm. Fluorescence and bright‐field images were overlaid to visualize the spatial distribution of SRB within the gel matrix.

### 4.13 In Vivo Drug Delivery Assessment

Male Sprague–Dawley rats (RRID:RGD_70508; *n* = 3), 7 weeks of age, were purchased from Daehan BioLink Co., Ltd. (Eumseong, South Korea). The animals were housed under standard laboratory conditions with free access to food and water. Before patch application, the dorsal hair was removed from the application area using an electric clipper. Sulforhodamine B‐loaded microneedle patches were applied to the depilated dorsal skin. As controls, untreated dorsal skin and dorsal skin treated with dye‐free (blank) R‐MAP‐ML patches were prepared in parallel under identical experimental conditions. To ensure microneedle insertion, the patches were manually pressed against the skin for 30 s and then secured with medical adhesive tape. The patches were maintained in contact with the skin for 12 h under normal housing conditions. After the 12 h application period, the patches were removed, and the treated skin area was gently wiped with PBS‐soaked gauze to remove residual surface dye. The dorsal skin was excised and embedded in OCT compound with the epidermal surface oriented perpendicular to the cutting plane. Serial cryosections were prepared at 30 µm thickness using a cryostat maintained at −23°C and mounted on glass slides. Fluorescence signals were immediately visualized using a DMi8 fluorescence microscope (Leica, Germany) equipped with a RHOD_LP filter set, with excitation at 535/50 nm and a long‐pass emission filter at 580 nm. Images were acquired using identical exposure time, gain, and illumination settings across samples.

### 4.14 Histological and Immunohistochemical Analysis

Paraffin blocks were sectioned at 4 µm and stained with haematoxylin–eosin (H&E) to screen for epithelial disruption (surface erosion or detachment) and submucosal inflammation. After heat‐induced epitope retrieval (EDTA pH 9.0 for pan‐cytokeratin; citrate pH 6.0 for all others), sections were blocked with 5% goat serum and incubated overnight at 4°C with the following primary antibodies: mouse anti‐pan‐cytokeratin AE1/AE3 (Thermo Fisher 53‐9003‐82, RRID:AB_2533494, 1:100), mouse anti‐CD163 2A10/11 (Bio‐Rad MCA2311GA, RRID:AB_322326, 1:100), mouse anti‐CD3ε PPT3 (SouthernBiotech 4510‐01, RRID:AB_2687920, 1:200), rabbit anti‐TNF‐α (Abcam ab6671, RRID:AB_305326, 1:200) and rabbit anti‐IL‐6 (Abcam ab6672, RRID:AB_305402, 1:400). Detection was performed with EnVision and HRP/DAB for pan‐cytokeratin, Alexa Fluor 488 goat anti‐mouse IgG (Thermo Fisher, A‐11001, RRID:AB_2534069, 1:500, 45 min) for CD3 and CD163, and Alexa Fluor 594 goat anti‐rabbit IgG (Thermo Fisher, A‐11037, RRID:AB_2534095, 1:500, 45 min) for TNF‐α and IL‐6; nuclei were counter‐stained with DAPI. Isotype‐matched IgG and “no‐primary” slides were negative. A porcine spleen section processed in parallel served as an external positive control for lymphoid markers (CD3, CD163), while scattered resident immune cells within normal oral mucosa provided internal reference staining for TNF‐α and IL‐6. Images were captured at ×400 using an Olympus BX53 (Tokyo, Japan) microscope equipped with a DP73 camera and cellSens software (Olympus, Tokyo, Japan).

### 4.15 Quantitative Image Analysis

High‐resolution immunofluorescence images were imported into QuPath v0.6.0 for quantitative analysis. From each of two pigs, oral mucosa was harvested at five untreated control sites and the anatomically matched five microneedle‐patch sites. One representative section per site was imaged under identical exposure settings. In every section, five non‐overlapping rectangular regions of interest (ROIs; 260 µm × 200 µm, 0.052 mm^2^) were placed in histologically equivalent zones by an operator blinded to treatment, avoiding folds, tangential cuts, or other artefacts. This yielded 50 ROIs per group (Control = 5 sites × 5 ROIs × 2 animals; Patch = same). Positive cells were identified with QuPath's “Cell detection” routine using empirically optimised nuclei‐ and cytoplasm‐based fluorescence thresholds plus marker‐specific size filters; all thresholds were fixed across the entire dataset. Detected cell counts from each ROI constituted individual data points. Distributions were assessed with Shapiro–Wilk tests; because normality was rejected (*p* < 0.05), medians (interquartile ranges) were compared between groups by the two‐tailed Mann–Whitney U‐test (α = 0.05).

### 4.16 Image Acquisition

Images were acquired using an Olympus BX53 fluorescence microscope (Tokyo, Japan) equipped with a DP73 digital camera and cellSens imaging software in semi‐automated mode. The fluorescence illumination system comprised an Olympus U‐LH100HG 100 W mercury burner powered by an Olympus U‐RFL‐T power supply. Imaging parameters were held constant throughout the study.

### 4.17 Ex Vivo Transdermal Drug Permeation Study

Ex vivo permeation of SRB from R‐MAP‐ML and dye‐loaded R‐MAP‐ML (1.0 cm^−2^) was evaluated using vertical diffusion cells (DHC‐6TD, Logan Instruments, Somerset, NJ, USA), adapted from established Franz‐cell methods based on the OECD Guideline for the Testing of Chemicals [56]. Briefly, human cadaver skin containing the epidermis and dermis (∼800 µm; UMC Science, Ilsan, Republic of Korea) was hydrated in PBS for 30 min and mounted with the stratum corneum facing the donor compartment. Each patch was applied to the skin and manually pressed for 30 s, after which the donor compartment was tightly sealed using Parafilm. The receptor compartment contained 6.0 mL PBS (pH 7.4) and was maintained at 32 ± 0.5°C under continuous stirring at 100 rpm. Independent diffusion cells were terminated at 0, 2, 4, 8, 12, 24, 48, and 72 h (*n* = 3 per time point), and the entire receptor medium was collected without replenishment. SRB fluorescence was measured using a GloMax Discover microplate reader (Promega, Madison, WI, USA) at excitation/emission wavelengths of 530/580 nm. Permeation was expressed as the cumulative receptor‐phase SRB fluorescence at each time point.

### 4.18 Epithelial Thickness Measurement

Epithelial integrity was quantified on H&E‐stained sections using FIJI/ImageJ v1.54 (calibration = 0.50 µm pixel^−1^). For each pig, two bilateral microneedle‐patch sites and their anatomically matched controls were analysed at four locations (buccal mucosa, tongue dorsum/ventrum, attached gingiva, hard palate). In every region, the epithelium was viewed with a 40×/0.75 NA objective; fields with folds, oblique cuts or processing artefacts were excluded. From each patch and control area, three equidistant, perpendicular transects were drawn from the outer epithelial surface to the epithelial–connective‐tissue junction, yielding 6 values per pig (3 patch + 3 control) and 12 per group when both animals were pooled. Across the four sites, this produced 24 patch and 24 control measurements per animal (*n* = 96 total). Two observers, blinded to treatment, followed a pre‐calibrated macro; inter‐observer concordance was excellent (intraclass correlation coefficient = 0.93). In cases of > 5% discrepancy, the section was re‐examined jointly. Normality of thickness distributions was confirmed with Shapiro–Wilk tests (*p* > 0.10); therefore, patch vs. control means were compared with unpaired, two‐tailed t‐tests for each site (α = 0.05). Results are visualised as Tukey box‐and‐whisker plots with individual data points overlaid in Figure 5b(ii).

## Author Contributions

J.L., Y.S., B.J.L., and S.B. conceived the project and designed the overall study. J.L., Y.S., Y.C., B.J.L., S.B., and D.K. developed the experimental methodology. J.L., Y.S., Y.C., and S.C. performed the experiments, and J.L., Y.S., D.K., H.K., D.Y., and J.P. conducted the animal experiments. J.L. and Y.S. performed the formal analysis, software‐based analysis, and data visualization. J.L., Y.S., B.J.L., S.B., and D.K. validated the experimental results and interpreted the findings. B.J.L., S.B., and D.K. provided resources, supervised the project, administered the project, and acquired funding. J.L. and Y.S. wrote the original draft. All authors reviewed and edited the manuscript and approved the final version.

## Funding

This work was funded by the Samsung Medical Center Grant (No. SMX1240961), the National Research Foundation of Korea (NRF) grants (Nos. RS‐2022‐NR072451, RS‐2024‐00343865, NRF‐RS‐2024‐00348648, and RS‐2024‐00404659), and the Ministry of Trade, Industry and Energy (No. 20025702).

## Conflicts of Interest

The authors declare no conflicts of interest.

## Supporting information




**Supporting File 1**: advs77211‐sup‐0001‐SuppMat.docx.

**Supporting File 2**: advs77211‐sup‐0002‐MovieS1.mp4.

**Supporting File 3**: advs77211‐sup‐0003‐MovieS2.mp4.

## Data Availability

The data that supports the findings of this study are available in the supplementary material of this article.
