# Supplementary material for: Root‐Inspired Microneedle Patch With Interlocked Rigid‐Soft Architecture for High Tissue‐Adaptiveness
Source: Adv Sci (Weinh). 2026 Aug 15:e77211. Online ahead of print. doi: 10.1002/advs.77211 (PMC13477245; doi:10.1002/advs.77211)
Supplement: Supplementary file 1 [file ADVS-9999-e77211-s002.docx]

Supporting Information

Root-Inspired Microneedle Patch with Interlocked Rigid-Soft Architecture for High Tissue-Adaptiveness

Jongchan Lee, Yeongjun Song, Yonghyun Cho, Hyoin Kim, Donghyun Youn, Jinbong Park, Seungbeom Choi, Dohyun Kwon*, Brian Jun Lee*, Sangyul Baik*

**Table S1.** Comparison of representative flexible/stretchable microneedle patch systems reported in recent literature and this work.

| Ref. | Fabrication  method | Main material of substrate | Material of Microneedle | Young's modulus of substrate material (MPa) | Young's modulus of microneedle material (MPa) | | E__microneedle_ /E__substrate_ | Wet adhesion |
| --- | --- | --- | --- | --- | --- | --- | --- | --- |
| This work |  | PDMS | BMF BIO Resin | 0.5 | 1614 | 3228 | | O |
| [21] | Wet etching | Parylene | Silicon / Gold | 3200 | 130000 | 40.625 | | X |
| [22] | Lithography | PDMS | PLGA | 3 | 1000 | 333.3 | | X |
| [23] |  | Polyimide | Epoxy (SU-8) / Gold | 2500 | 2000 | 0.8 | | X |
| [24] |  | PET Film | CMRF (Magnetic Fluid) | 2000 | 1000 | 0.5 | | X |
| [25] |  | PET Film | CMRF | 2000 | 1000 | 0.5 | | X |
| [26] |  | PDMS / PEG-DA | PEG-DA | 3 | 20 | 6.67 | | X |
| [27] |  | PDMS | Epoxy (SU-8) | 3 | 2000 | 666.6667 | | X |
| [28] | 3D Printing | PLA | PLA | 3000 | 3000 | 1 | | X |
| [29] |  | Stretchable / Elastomeric Resin | Rigid Resin | 10 | 1500 | 150 | | X |
| [30] | Assembly | OSTEMER (Thiol-ene-epoxy) | Stainless Steel | 15.5 | 180000 | 11612.9 | | X |
| [31] |  | SEBS | Cellulose Acetate | 1.5 | 1800 | 1200 | | X |
| [32] | Micromolding | PET Film | PLGA / GelMA | 2000 | 500 | 0.25 | | X |
| [33] |  | PVA | Hydrolyzed Collagen | 3000 | 2300 | 0.767 | | X |
| [34] |  | PVA / PVP | PVP | 3000 | 3000 | 1 | | X |
| [35] |  | PEG-PEGDA | PEG-PEGDA | 20 | 20 | 1 | | X |
| [36] |  | SF-PU | SF-PU + AG NWs/SF | 200 | 750 | 3.75 | | X |
| [37] |  | PET Film | PVP-CPO | 2000 | 3000 | 1.5 | | X |
| [38] |  | NOA 81 | PEGDMA / NVP / DMAEA / 3APBA | 1380 | 300 | 0.217 | | X |
| [39] |  | PEGDA | PEGDA / Sucrose / SF | 20 | 750 | 37.5 | | X |
| [40] |  | PLA | PLGA | 3000 | 1000 | 0.333 | | X |
| [41] |  | Polyimide | Polyimide /Gold/PEDOT:PSS | 2500 | 2500 | 1 | | X |
| [42] |  | PVA | SF | 3000 | 750 | 0.25 | | X |
| [43] |  | HA | HA | 100 | 100 | 1 | | X |
| [44] |  | HA | HA | 100 | 100 | 1 | | X |
| [45] |  | HA | HA + TiO2-x nanoparticle | 100 | N/A | N/A | | X |

**Figure S1.** a) Schematic illustration of the array exposure mechanism, in which a moving light engine enables step-and-repeat projection for large-area fabrication. b) Schematic illustration of the various printing mode of microArch S130.

**Figure S2**. a) Experimental setup for peel-off testing used to evaluate interfacial adhesion between 3D-printed microneedle base molds and PDMS substrates. The interlocking force resulting from the structural integration of the PDMS and lattice, as well as the fracture force at the lattice–microneedle junction, are measured. b) Raw force–displacement curves obtained from peel-off tests. c) Representative images of peel-off test outcomes showing successful and unsuccessful interlocking between the PDMS and lattice structure.

**Figure S3**. Interlocking strength of various biocompatible elastomers with the lattice structure.

**Figure S4**. Quantitative analysis of Young’s modulus and flexural stress of R-MAPs as a function of microneedle spacing ratio. Increased SR is associated with reduced stretchability and flexibility of the patch.

**Figure S5**. Representative stress–strain curve obtained from uniaxial tensile testing of the R-MAP. The patch was stretched at a rate of 5 mm min⁻¹ with both ends clamped.

**Figure S6**. (i) Optical images of the stretchability test of the R-MAP. (ii) Optical image of the R-MAP under 180° torsional deformation. (iii) Raw data obtained from cyclic tensile loading tests.

**Figure S8**. a) Representative stress–strain curve obtained from uniaxial tensile testing of the R-MAP. The patch was stretched at a rate of 5 mm min⁻¹ with both ends clamped. b) NHS group forms covalent cross-linking through a nucleophilic attack by the lone pair electrons on the amine group on buccal tissue.

**Figure S7**. SEM images root structure of root-inspired microneedle varying cell size. a) 70 µm and b) 100 µm (Scale bar = 300 µm).

**Figure S9.** Through oxygen plasma treatment of the PDMS substrate, the surface is modified to be hydrophilic, and benzophenone treatment enables covalent bonding with the hydrogel via its role as a photo‐initiator in UV‐mediated free radical polymerization. Upon application of the ML precursor onto the R-MAP surface followed by UV exposure, a thin mucoadhesive layer is uniformly formed on the surface of the R-MAP.

**Figure S10**. FT-IR spectrum of the mucoadhesive layer (ML) of R-MAP-ML was analyzed to identify its chemical functional groups. Specific peaks observed at 1,158 and 1,209 cm⁻¹ are attributed to the C-N-C stretching vibrations within the NHS ester groups. Additionally, the characteristic peak at 1,696 cm-1 confirms the presence of C=O stretching from the carboxylic acid groups in PAA.

**Figure S11.** a) Normal adhesion test. The patch was applied onto ex vivo porcine oral mucosal tissue under a constant preload of 30 kPa for 1 min, followed by perpendicular detachment at a rate of 3 mm s⁻¹ using a universal testing machine. b) Shear adhesion test. The patch–tissue interface was compressed under the same preload (30 kPa), and lateral displacement was applied at 3 mm s⁻¹ until adhesive failure, simulating frictional stresses encountered intraorally. c) Peel-off adhesion test. The edge of the patch was gripped and peeled from the tissue at an angle of 90° relative to the surface, under a constant peeling speed of 3 mm s⁻¹ after 30 kPa preload application. These three complementary setups enabled systematic evaluation of R-MAP adhesion robustness under distinct loading modes representative of oral environments.

**Figure S12**. Schematic design of a single-axis friction device for ex vivo adhesion testing.

**Figure S13.** Sequential photographs of the in-vivo adhesion assessment procedure at the target site, showing 20 s preloading, adhesion, and 100 rubbing cycles.

**Figure S14.** Representative histological image of porcine oral tissue after R-MAP penetration.

**Figure S15.** In vitro microneedle penetration and sulforhodamine B delivery in agarose gel.

a) Bright-field image showing penetration of the R-MAP microneedle into agarose gel. b) Fluorescence image showing sulforhodamine B distribution in the agarose gel after microneedle insertion.

**Figure S16.** Fluorescence microscopy images of cryosectioned rat dorsal skin demonstrating Sulforhodamine B delivery via R-MAP-ML after 12 hours application (n = 3). (i) Fluorescence and (ii) brightfield images of control tissue showing no detectable fluorescence signal. (iii) Fluorescence and (iv) brightfield-fluorescence overlay images of R-MAP-ML-treated tissue, revealing concentrated sulforhodamine B signals (red) localized at discrete sites corresponding to microneedle insertion points. Spatial colocalization of dye accumulation with regions of epithelial disruption in the overlay confirms transepidermal compound delivery through microneedle-created penetration pathways. Scale bar = 750 µm.

**Figure S17.** R-MAP-ML without a hydrogel coating and dye-loaded R-MAP-ML were applied to human cadaver skin mounted in vertical diffusion cells. SRB permeation into the receptor compartment was assessed by fluorescence measurement over 72 h. Values were normalized to the predefined reference fluorescence value. Data are presented as the mean ± SEM of three independent diffusion cells at each time point.

**Figure S18.** Representative immunohistochemical staining of pan-cytokeratin (PanCK) in buccal mucosa from control and microneedle patch–treated sites. Both groups exhibited continuous and uniform epithelial staining without evidence of surface erosion, epithelial detachment, or cytokeratin loss (scale bar = 50 µm).
